# Audiology Workforce Supply and Population Demand: Trends, Benchmarks, and Implications

**DOI:** 10.1055/s-0046-1825792

**Published:** 2026-10-06

**Authors:** Victor Bray, Amyn M. Amlani

**Affiliations:** 1 Osborne Audiology Department, College of Nursing and Health Professions, Drexel University, Elkins Park, Pennsylvania, United States; 2 Department of Communication Sciences and Disorders, College of Allied Health, University of Oklahoma Health Sciences Center, Oklahoma City, Oklahoma, United States

**Keywords:** audiology workforce, workforce supply and demand, hearing healthcare access, professional attrition, hearing aid specialists

## Abstract

Audiology's viability as a self-sustaining, autonomous profession depends on a workforce capable of meeting population demand. This article benchmarks audiology's (AuD) workforce supply against comparable healthcare professions and projected growth in hearing loss prevalence. Using Bureau of Labor Statistics data from 1999 to 2024, AuD workforce trends were compared with 16 other advanced-degree healthcare professions. AuD's mean annual growth rate (1.77%) ranked third lowest among 17 master's- and doctoral-level professions, well below four peer disciplines (occupational therapy, physical therapy, optometry, speech-language pathology), which grew 5.5 times faster over the same period. An input/output model of graduate production versus workforce departure showed that AuD's constrained growth stems not from insufficient educational throughput but from elevated departure rates, including abandonment of the profession. Meanwhile, the hearing aid specialist workforce grew over 200% between 2012 and 2024, suggesting substitution in core clinical functions. Population modeling projects the number of U.S. adults with hearing impairment will nearly double by 2060, widening the supply-demand gap. These findings indicate that AuD's transition to a doctoral entry-level degree introduced a lasting structural constraint on workforce capacity, with implications for access to care, professional autonomy, and audiology's positioning within value-based healthcare delivery.

## Introduction

In the preface of this special issue, we asserted that a viable workforce is a prerequisite for audiology to function as a free-standing, autonomous profession. A viable workforce is fundamental to ensuring access to quality healthcare 1 and serves as a critical resource for health systems seeking to expand and modernize service delivery. 2 Without sufficient workforce capacity, even clinically effective interventions cannot be deployed at scale.

This article benchmarks audiology's capacity to supply providers against population demand and compares its workforce growth to that of peer healthcare professions. Despite producing a steady pipeline of doctoral-level graduates, audiology has not translated educational output into sustained workforce growth under current economic and regulatory conditions. As demonstrated in this analysis, audiology's occupational expansion lags substantially behind that of comparable healthcare professions, including those with lower terminal degree requirements. This mismatch between training intensity and workforce return represents a structural vulnerability—one that constrains access to care, undermines professional autonomy, and threatens audiology's long-term positioning within a value-based healthcare system.

The findings presented here have direct implications for audiology leadership, professional organizations, and academic institutions responsible for shaping workforce and policy decisions. Addressing these structural constraints is essential for audiology to meet rising population demand and maintain its relevance as a doctoring health profession.

## Workforce Supply by Healthcare Occupation

To benchmark audiology's workforce trajectory, we analyzed Bureau of Labor Statistics (BLS) Standard Occupational Classification (SOC) data using the Occupational Outlook Handbook/Healthcare Options across healthcare occupations with comparable educational requirements. 3 We then combed through the dataset and removed professions that did not meet the following criteria: (a) was not listed with a 29-xxxx SOC (i.e., not healthcare), (b) did not require a college degree, (c) were in categories of physicians and surgeons, or (d) did not have a complete dataset spanning between 2012 and 2024. Following this exercise, 31 occupations were identified, of which 9 required associate degrees, 5 required bachelor's degrees, 9 required master's degrees, and 8 required doctoral or professional degrees.

It is important to note that the BLS occasionally reviews and revises their SOC professions and tables, and this impacts some of the information presented in this manuscript. In 1999, audiology (AuD) and speech-language pathology (SLP) were entered as separate professions and 1999 serves as the base year for Figs. 3 and 5 . In 2012, the BLS added several more SOC professions and 2012 serves as the base year for Figs. 1 and 2 . BLS data are available through 2024, which serves as the end year for Figs. 1 , 2 , 3 , and 6 . Also, Figs. 1 , 2 , and 4 are updated with revised methodology from a prior publication in *Audiology Practices* . 4 Table 1 5 , and Table 2 and Fig. 6 6 are revised from previous poster presentations.

**Fig. 1:**
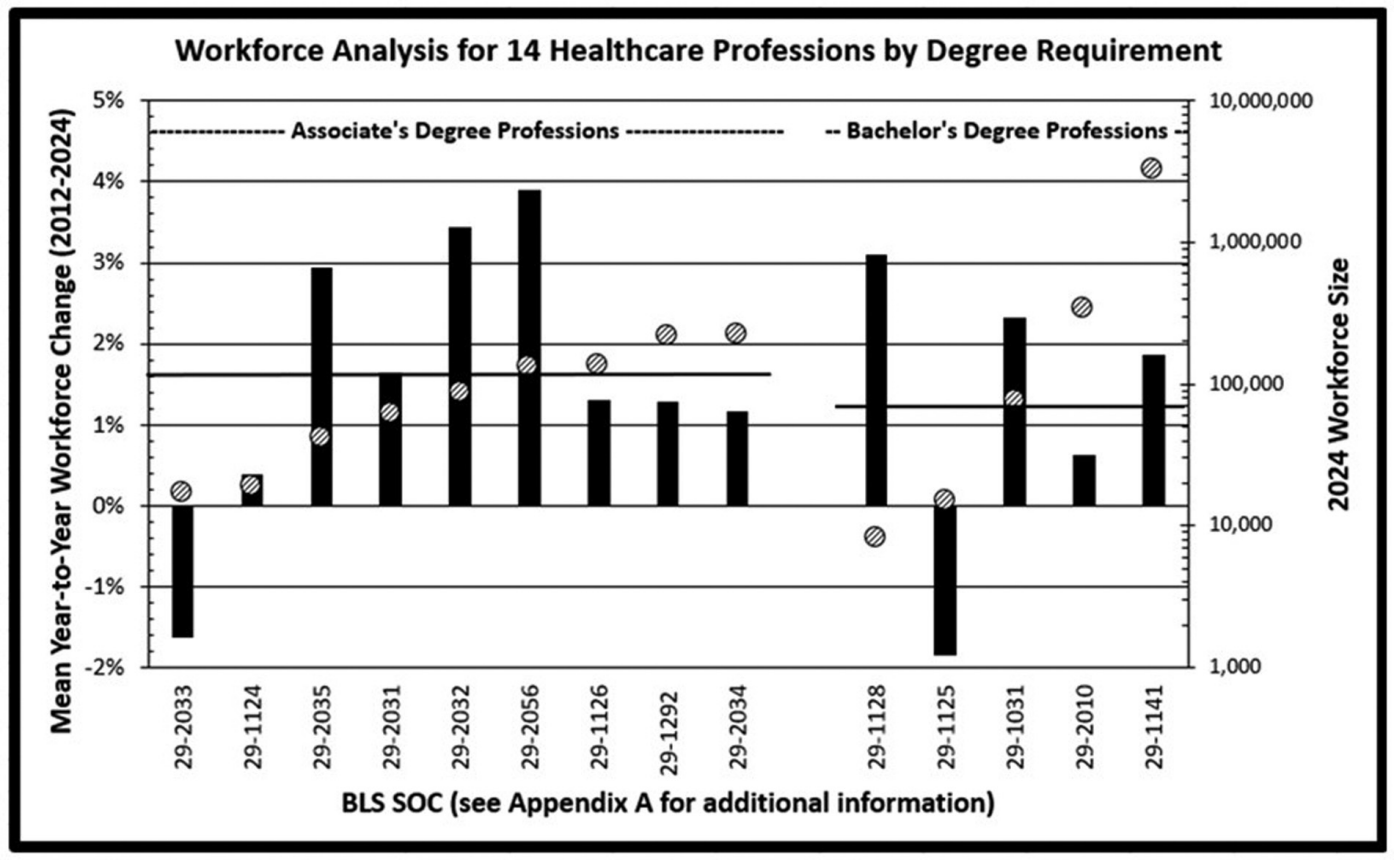
Comparative changes in workforce (2012–2024). Workforce analysis among healthcare professions as reported by degree designation (associate's, bachelor's) between 2012 and 2024. Professions are identified on the abscissa using the Bureau of Labor Statistics (BLS) Occupational Classification (SOC) system. The linkage between BLS SOC and profession name is shown in Appendix A . The left ordinate is the mean year-to-year change in each profession's workforce from 2012–2013 through 2023–2024 and is displayed with vertical bars. The right ordinate is the professional workforce size in 2024 and is displayed as circles. The two horizontal bars indicate the mean percentage change within a profession's degree category, left side for associate's degree and right side for bachelor's degree.

**Fig. 2:**
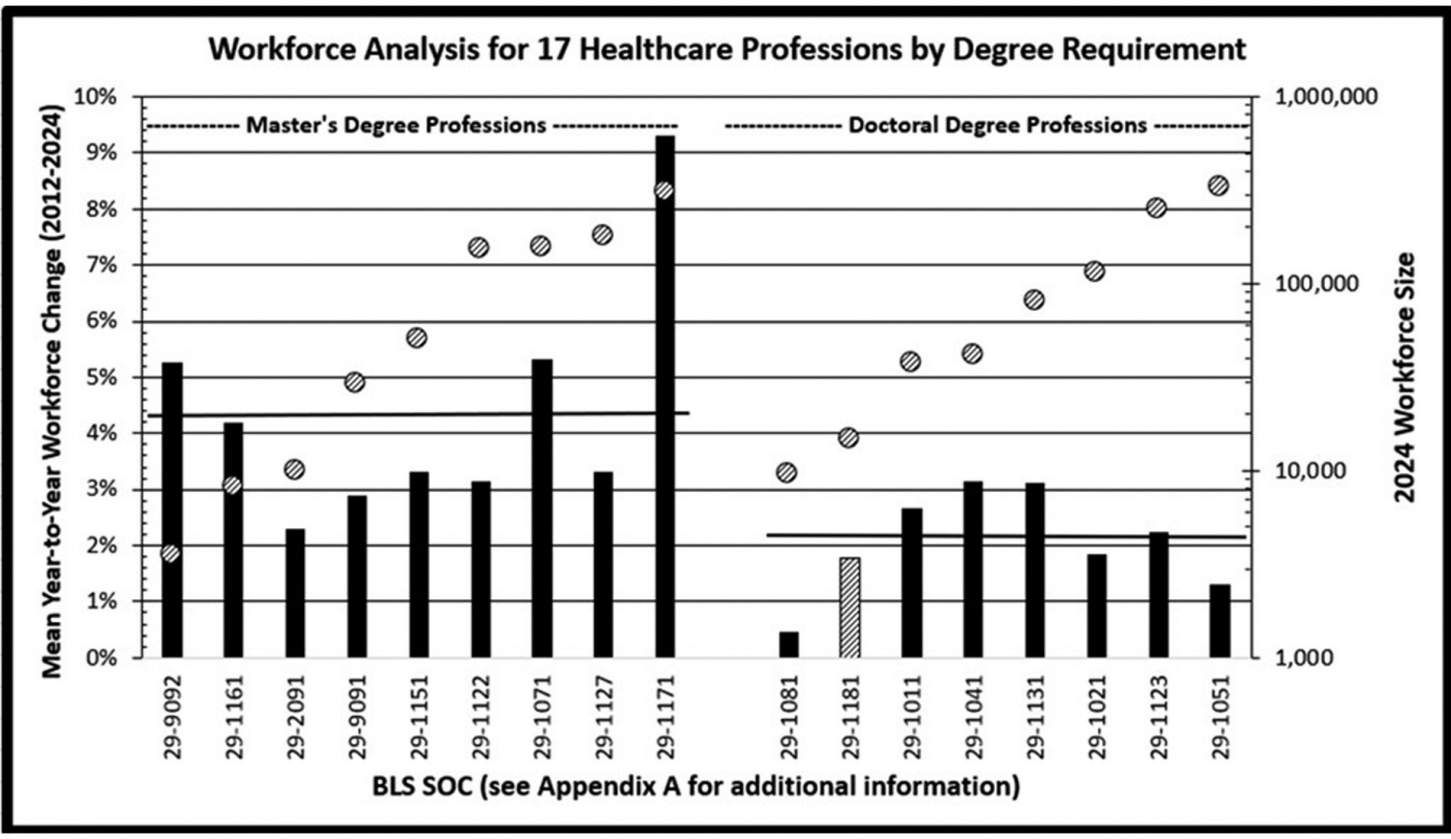
Comparative changes in workforce (2012–2024). Workforce analysis among healthcare professions as reported by degree designation (master's, doctoral) between 2012 and 2024. Professions are identified on the abscissa using the Bureau of Labor Statistics (BLS) Occupational Classification (SOC) system. The linkage between BLS SOC and professional name is shown in Appendix A . The left ordinate is the mean year-to-year change in each profession's workforce from 2012–2013 through 2023–2024 and is displayed with vertical bars. Profession 29–1181 (audiology) is highlighted with the striped horizontal bar. The right ordinate is the professional workforce size in 2024 and is displayed as circles. The two horizontal bars indicate the mean percentage change within a profession's degree category, left side for master's degree and right side for doctoral degree.

**Fig. 3:**
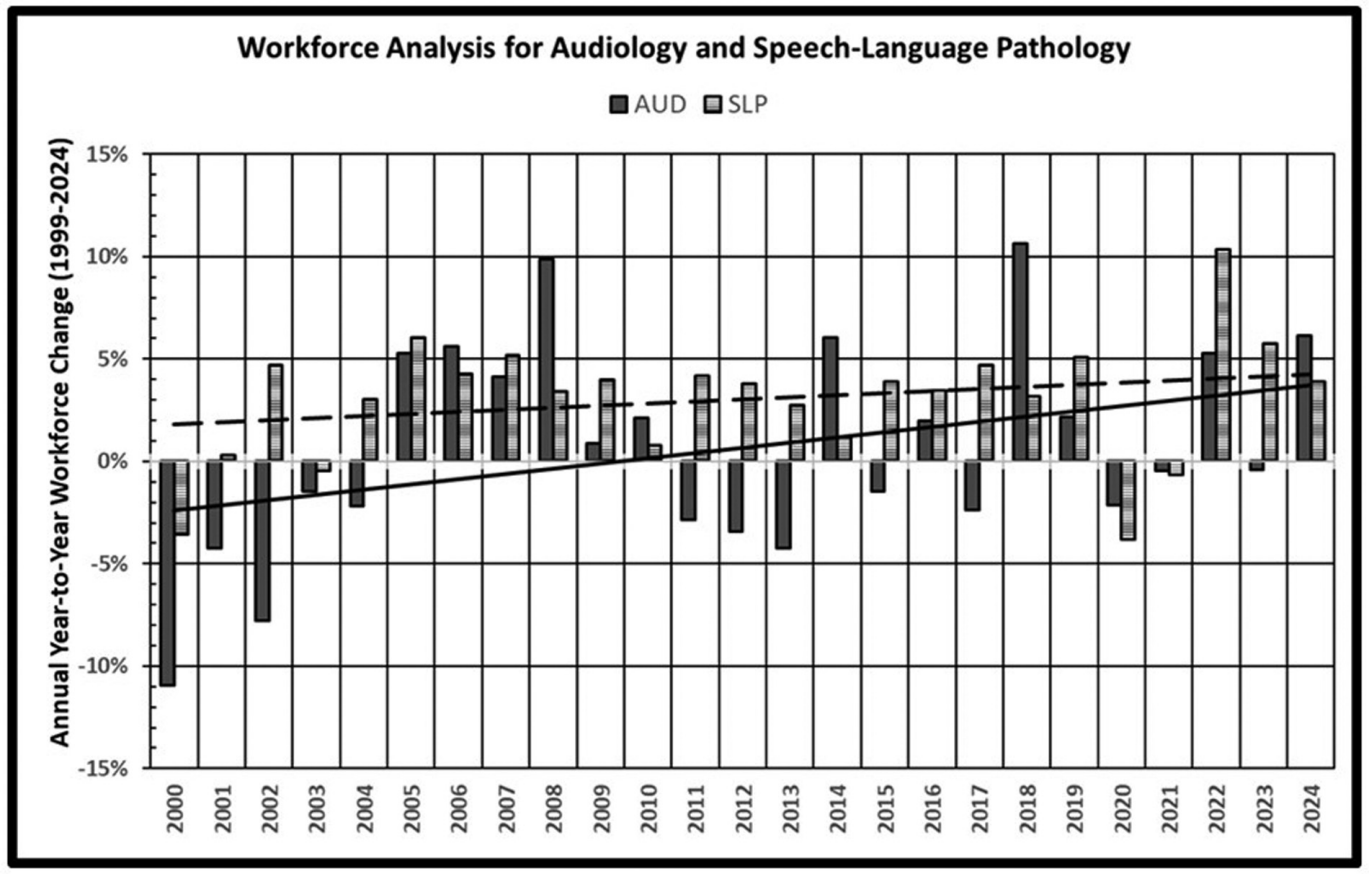
Comparative changes in workforce (2000–2024). Workforce analysis comparing audiology (AuD) and speech-language pathology (SLP) from 2000 to 2024. The left ordinate is the mean year-to-year change in each profession's workforce from 1999–2000 through 2023–2024 and is displayed with vertical bars. The solid line represents linear regression analysis for AuD and the dashed line represents linear regression analysis for SLP.

**Fig. 4:**
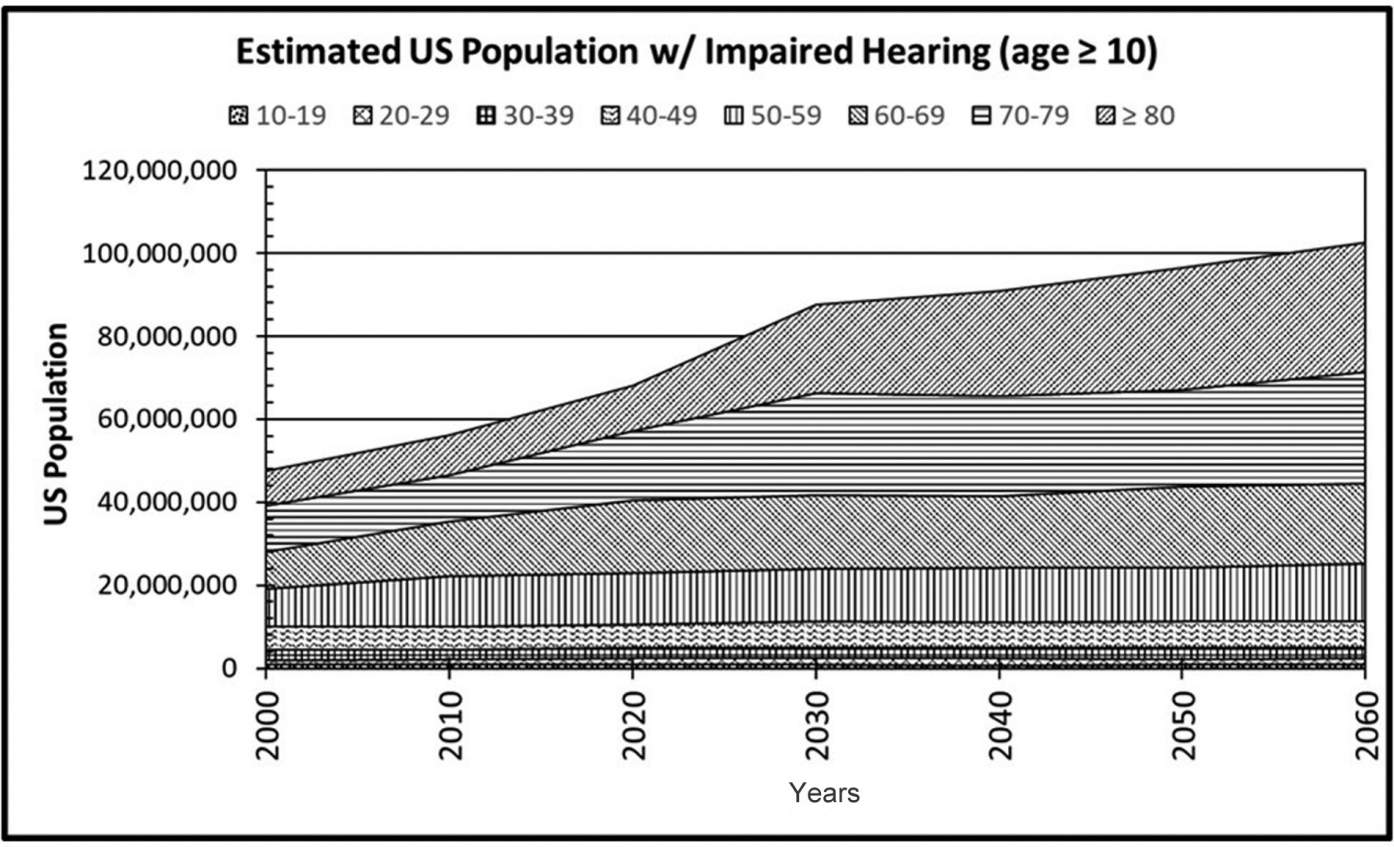
Estimated number of people in the United States with impaired hearing in one or two ears (ages ≥ 10 years) between 2000 and 2060. The figure was created by combining US population by age 10 and US hearing impairment by age. 11

**Fig. 5:**
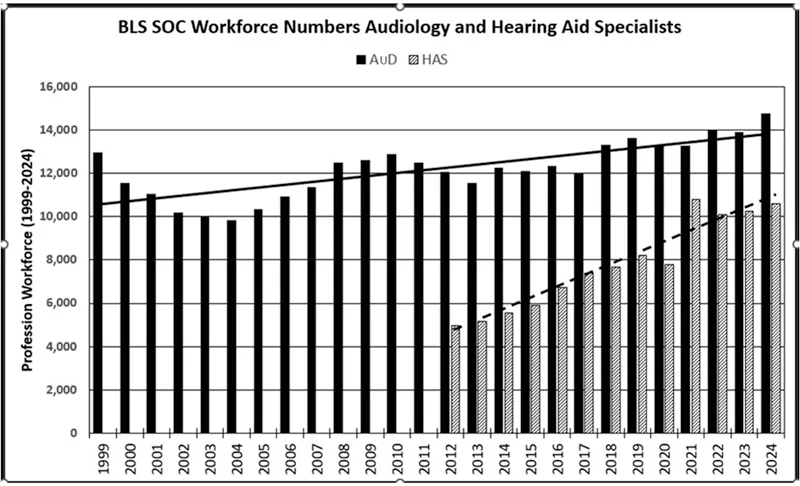
Workforce analysis comparing audiology (AuD) and hearing aid specialists (HAS). Bureau of Labor Statistics (BLS) data for AuD begins in 1999 and for HAS in 2012. The abscissa is the reported workforce year, and the ordinate is the reported workforce size. Two linear regression lines are shown, the solid line for AuD and the dashed line for HAS.

**Fig. 6:**
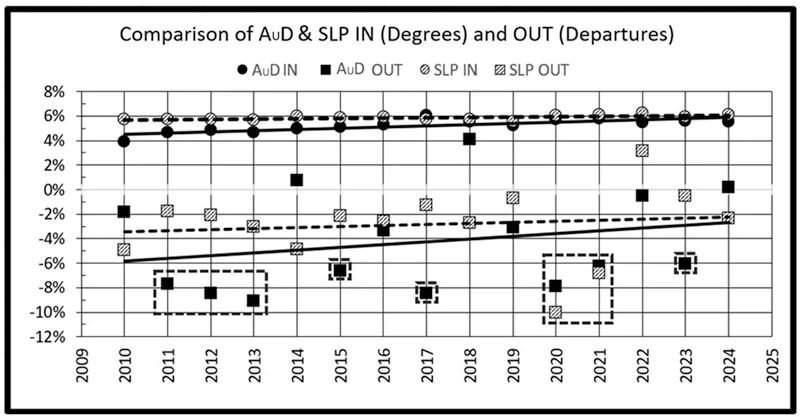
Scatterplot (2009–2024) for audiology (AuD) and speech-language pathology (SLP) for AuD IN and SLP IN (degrees/workforce) and AuD OUT and SLP OUT (change minus degrees/workforce). Linear regression lines for AuD are solid and dotted for SLP. For AuD OUT, several data points are surrounded by a dashed box. These identify years where the OUT rate (%) was greater than the IN rate (%). The large dashed box for 2020 and 2021 identifies AuD OUT and SLP OUT that occurred during the COVID-19 pandemic.

**Table 1:** Profession growth rate: comparison of workforce growth among five comparable healthcare professions

	Audiology	Occupational therapy	Optometry	Physical therapy	Speech-language pathology
1999/2000	12,240	77,050	22,640	125,730	84,385
2023/2024	14,305	148,560	41,640	244,725	175,445
Growth	16.9%	92.8%	83.9%	94.6%	107.9%

Note: The base value is the average of reported workforce for 1999 and 2000. The current value is the mean of the reported workforce for 2023 and 2024. The percentage growth represents the change from the base years to the current years.

**Table 2:** Population analysis for estimated demand (persons with hearing impairment, HI) versus estimated supply (audiologists, AuD) from 2000 through 2060

	2000	2010	2020	2030	2040	2050	2060
Total US population (≥10 years)	243.2M	269.3M	300.1M	330.1M	331.7M	340.4M	350.4M
Population with HI (≥10 years)	47.4M	56.1M	68.2M	87.6M	90.8M	96.3M	102.5M
% population with HI (≥10 years)	19.5%	20.8%	22.7%	26.5%	27.4%	28.3%	29.3%
Audiologists (BLS)	11,530	12,860	13,300	15,683	17,963	20,243	22,522
HI population (≥10 years)/AuD	4,111	4,362	5,128	5,586	5,055	4,757	4,551

Abbreviation: BLS, Bureau of Labor Statistics.

Note: US population data are actual through 2020 and are estimated beginning in 2030. Audiologists data are actual through 2020 (BLS) and estimated beginning in 2030 using the growth rate extracted from Fig. 6 (2012–2024).

It is also important to understand that the BLS tables do not capture all people within a profession. For example, the BLS Occupational Employment and Wage Statistics (OEWS) reports 14,730 audiologists in the workforce for 2024, but their estimate does not include self-employed workers. 7 In contrast, the BLS Occupational Outlook Handbook reports the size of the profession as 15,800 audiologists. 8 Throughout this paper, the BLS OEWS tables are used for workforce numbers. The inherent value of using the OEWS tables is they are available from 1999 and provide a standardized method to compare AuD to other professions in benchmarking using longitudinal analysis. However, the numbers presented from the BLS OEWS tables do represent a slight undercount of workforce sizes of the professions.

Fig. 1 displays the mean year-to-year change in workforce among healthcare occupations requiring an associate degree or bachelor's degree over a 13-year span (i.e., 2012–2024). The mean growth rate for the associate's degree professions was 1.6 and 1.2% for bachelor's degree professions. One associate's degree profession, 29–2033 (nuclear medicine technologists), displayed negative mean year-to-year growth, as did one bachelor's degree profession, 29–1125 (recreational therapists). Two associate's degree professions displayed greater than 3% mean year-to-year growth, 29–2032 (diagnostic medical sonographers) and 29–2056 (veterinary technologists and technicians), as did one bachelor's degree profession, 29–1128 (exercise physiologists).

Fig. 2 displays the mean year-to-year change in workforce among healthcare occupations requiring a master's degree or doctoral degree over a 13-year span (i.e., 2012–2024). The mean growth rate for the master's degree professions was 4.3 and 2.1% for doctoral degree professions. No professions displayed negative year-to-year growth rates. Three master's degree professions displayed greater than 5% mean year-to-year growth, 29–9092 (genetic counselors), 29–1071 (physician assistants), and 29–1171 (nurse practitioners). Two doctoral degree professions displayed greater than 3% mean year-to-year growth, 29–1041 (optometrists) and 29–1131 (veterinarians). The mean year-to-year growth rate for AuD (29–1181) was 1.77%, the third lowest of the 17 advanced-degree professions' profiles.

As shown in Figs. 1 and 2 , across healthcare occupations, workforce growth varies substantially by degree by degree requirement and by profession. The highest group workforce growth rate was in the master's-level professions and 29–1171 (nurse practitioners), the largest within this category. All nine of the master's degree professions grew at faster rates (range 2.3 to 9.3%) than AuD (1.8%). This comparative context is essential for interpreting AuD's trajectory, in moving from a master's degree to doctoral-level training. As part of the interpretation, it is important to note that another profession that transitioned from master's to doctoral degree, 29–1123 (physical therapy), grew 2.2% annually, which was greater than the mean group performance for doctoral degree professions.

## Audiology Occupational Insights

In this section, we examine occupational growth in AuD. In 2019, 9 we reported that AuD's workforce increased by only 5% between 1999 and 2019, a period during which other healthcare professions expanded by 146 to 203%, and overall healthcare employment grew by 46%. This divergence indicates that AuD's workforce trajectory is not only slower than that of peer professions, but qualitatively different in its pattern of expansion. To refine this observation, subsequent analyses applied linear regression methods to the same dataset to characterize underlying growth trends. 4 These analyses yielded an estimated annual workforce increase of approximately 94 audiologists, corresponding to a 20-year growth of 17.3%.

Historically, AuD has been thought of as an allied health profession, on par with occupational therapy, physical therapy, and SLP. As part of the AuD-transition movement, it was hoped by many that in the future the profession would be considered on par with optometry. The parity basis would be that both professions had 4-year doctoral degree programs, were focused on primary sense disorders, and had scopes of practice to evaluate, diagnose, treat, and manage patients within the respective areas of professional expertise. Given this cluster of four professions with widely accepted similarities to AuD, data were extracted from BLS OEWS tables to generate another benchmark closer to home.

Table 1 reveals that over the period from 1999/2000 to 2023/2024, the AuD workforce grew by approximately 17%, in contrast to the mean growth of 95% for the four similar professions. The fact that the other four professions grew at a rate of 5.5 times the AuD rate reinforces the conclusion that net workforce accumulation in AuD has been severely limited over the past 25 years.

A basic observation from repeated analyses of the BLS tables is that healthy healthcare professions can be expected to approximately double in workforce size over a 25-year period. This lack of appreciable growth in AuD is a complex issue to untangle, and it is recommended that the interested reader see the accompanying manuscripts in this special issue on AuD profession of *Seminars in Hearing* .

Fig. 3 shows the year-to-year workforce change for the two communication sciences and disorders (CSD) professions (AuD and SLP), from 1999–2000 to 2023–2024. Using annual change data, the mean growth in AuD workforce was 0.65% (standard deviation of 5.20%) and 3.02% (standard deviation 3.05%) for SLP. It is interesting that this 4.6 faster growth rate of SLP over AuD is largely occurring inside the same academic units (CSD) and accreditation body (Council for Academic Accreditation for Audiology and SLP).

Over the 25 years reported in Fig. 3 , AuD displayed negative growth in 13 (52%) of the years and SLP had negative growth in four (16%) of the years. Two periods of marked negative workforce growth in AuD include 2000–2004 (cumulative change of −27%) and 2011–2013 (cumulative change of −11%). This pattern continues even today, with negative growth occurring in 2020, 2021, and 2023. The linear regression lines in Fig. 3 show a long-term change from −2.5% (2000) to just under +4% (2024) for AuD and from +2% (2000) to just over +4% (2024) for SLP.

As shown in Fig. 3 , these trends reflect a pronounced structural discontinuity in AuD's workforce history. Unlike gradual market-driven fluctuations observed in other health professions, this contraction represents a discrete workforce shock associated with educational restructuring. Although workforce recovery began in 2005 and showed a period of cumulative growth of 28% through 2010, this brief recovery was not maintained and has not fully offset the cumulative workforce deficit introduced during the transition period.

Because of the volatility in the AuD workforce numbers, estimates of AuD's annual workforce growth vary depending on the analytic window examined. Long-horizon and endpoint analyses yield annualized growth estimates of approximately 110 audiologists per year (1999–2021), whereas post-transition and more recent trend-based analyses yield higher estimates ranging from approximately 152 (2004–2021) to 200 audiologists per year (2012–2021). However, even at the upper bound of this range, workforce growth remains insufficient to compensate for earlier losses or to keep pace with projected population demand and peer healthcare professions. 4

Taken together, these findings suggest that the transition to the AuD as the terminal degree introduced a structural legacy effect on workforce capacity rather than a transient disruption. AuD's current workforce trajectory, while improved but not yet stable, reflects constrained expansion that continues to shape the profession's inability to meet growing demand for services.

### Application of Findings to Patient Care

Fig. 4 illustrates projected growth in the U.S. population with hearing loss across age cohorts, highlighting the demographic forces driving increased demand for audiologic care. In Fig. 4 , the estimated number of persons with hearing impairment, in one or two ears, is graphed in year decade intervals from 2000 through 2060. Individual bands are captured in age decade intervals from 10–19 through ≥80. In general, the numbers show a transition from approximately 50 million in 2000 to 100 million in 2060. This estimated doubling of the number of people with hearing impairment results from (a) hearing loss correlation with aging, (b) demographic shifts toward an older population, and (c) a growing population.

Table 2 displays information used to estimate the number of people (10 years of age or older) with impaired hearing who will need to be provided care by an audiologist. Of particular interest is that, due to demographic changes, the rate of the hearing impaired population grows faster than the rate of growth of the population. The estimates are, within this age group, the percentage of the population with impaired hearing will grow from 19.5% in 2000 to 29.3% in 2060. This suggests a dramatic 50% increase in potential need for audiological services over the 60-year period.

Fig. 4 , combined with Table 2 , illustrates one consequence of the failure to increase the AuD workforce at the rate needed to match the growth of people with hearing impairment. In this modeling shown in Table 2 , in 2000 there was 1 audiologist per approximately 4,000 potential patients. As population aging accelerates, this ratio worsens markedly under current supply trajectories. This increased in 2020 to 1 audiologist per approximately 5,000 potential patients, and in 2030 peaks at 1 audiologist per approximately 5,500 potential patients. With growth in the AuD workforce, combined with changing demographics (see reduced overall slope for total HI after 2030 in Fig. 4 ), by 2060 the ratio may improve to 1 audiologist per approximately 4,500 potential patients. These ratios underscore a growing and long-term capacity gap in hearing healthcare delivery that cannot be addressed solely through incremental workforce growth.

The reader must interpret Table 2 cautiously, as it underestimates potential patients (it does not include pediatric patients [≤10] with hearing impairment or vestibular and tinnitus patients of any age), and it underestimates the workforce (as is known when using the BLS database). Nonetheless, the message is clear on emerging trends of concern regarding demand for services.

In parallel, BLS data indicate that workforce growth among hearing aid specialists (HAS; SOC 29–2092) has accelerated substantially, exceeding 200% between 2012 and 2024. Fig. 5 compares the workforce numbers for AuD (1999–2024) and for HAS (2012–2024). For AuD, the regression line slope for 1999 to 2024 shows average year-to-year workforce growth of 130 per year. In a parallel analysis (not shown), the AuD regression line slope for 2012 to 2024 represented workforce growth of 228 per year. In contrast, the regression line for HAS (2012–2024) yielded an average growth rate of 519 per year. If these two professions continue with these occupational growth rates, where the smaller profession is growing at more than two times the larger profession, the situation will reverse and the smaller profession will become the larger profession around 2036. This crossover is not merely a numeric milestone; it signals a shift in market-scaled hearing care capacity that may increasingly shape access pathways, service delivery models, and scope-of-practice competition within the hearing healthcare ecosystem. Emerging market data suggest that this transition is not incidental—AuD practice owners are increasingly abandoning third-party administrator (TPA) contracts, with HAS providers absorbing those patient relationships and the revenue associated with them; the economic implications of this substitution dynamic are examined in detail in Article 7 (Health Economics) of this issue.

It should also be noted that the entry-level clinical skills for audiologists are to produce audiograms and fit hearing aids, which is also the scope of practice for HAS. Extrapolating the data in Table 1 , if AuD growth had matched its peer professions, there would be approximately 24,000 audiologists in the 2024 workforce. Because the data show a little over 14,000 audiologists in 2024, the question arises who is doing the work of the missing 10,000 audiologists to test hearing and fit hearing aids? From Fig. 5 , one can see the answer: in the absence of growth in the AuD workforce, there is rapid growth in the HAS workforce. If the 2024 workforce numbers are added for the two professions, a joint workforce of approximately 24,000 results. This begs the question as to whether the nation's healthcare system, in general, values audiologists other than a focus on fitting and dispensing hearing aids.

### Health of the Audiology Workforce

Basic workforce modeling is built on three factors: input to workforce, current workforce size, and departure from the workforce. For a profession to grow, input must exceed departure and the greater the ratio of input to departure, the faster the profession grows. To investigate input, Bray and Amlani 5 examined the AuD degree pipeline relative to 10 peer healthcare professions. Data on graduate degree production for AuD and comparator professions were obtained from publicly available reports published by the respective accrediting bodies and professional associations (see Appendix B ). These data were used to derive graduate-to-workforce ratios and were not intended to establish absolute workforce counts. Together, these metrics provide a measure not only of workforce growth, but of the profession's capacity to convert educational output into sustained clinical supply.

Table 3 displays data from 10 professions across 7 years, with the percentage value of the number of graduate degrees awarded for the profession divided by the workforce size for that year. The mean value across the 7 years for each profession is shown in the column labeled Mean. The average of the values in the Mean column is 6.5% for the 10 professions and 6.0% for the 8 professions when the two extreme values are removed (14.5% for nurse practitioners and 2.5% for orthotists and prosthetists). The mean value for AuD was found to be 5.7% and near the mean values (6.0–6.5%) for the group.

**Table 3:** Graduate-to-workforce ratio (2017–2023) (Graduate degrees awarded by year for 10 advanced-degree healthcare professions [from Appendix B ] as a percentage of the reported Bureau of Labor Statistics [BLS] workforce for that year.)

	2017	2018	2019	2020	2021	2022	2023	Mean
Nurse practitioner	15.6	16.0	15.0	17.0	15.3	12.0	10.7	14.5
Chiropractic	7.2	7.8	7.6	8.3	8.3	7.8	7.0	7.7
Physician assistant	7.6	8.0	7.9	N/A	6.2	5.8	5.5	6.9
Occupational therapy	5.5	6.0	5.8	6.1	8.2	6.5	5.9	6.3
Speech-language pathology	6.0	5.8	5.8	6.1	6.9	6.3	6.3	6.2
Podiatry	6.0	5.8	6.0	5.8	6.2	6.1	6.0	6.0
Audiology	6.1	5.4	5.6	5.8	5.8	5.6	5.9	5.7
Physical therapy	4.6	4.6	4.5	5.1	5.4	5.1	5.0	4.9
Optometry	4.5	4.5	4.4	4.7	4.7	4.3	4.4	4.5
Orthotists and prosthetists	N/A	N/A	2.2	2.6	2.3	2.7	2.9	2.5

Abbreviation: N/A, data not available.

In the interpretation of Table 3 , it would seem to be a fair conclusion that higher graduate-to-workforce ratios correlate with higher workforce growth rates. However, this conclusion is only valid if all professions have similar retention (e.g., practice longevity) rates. Although the AuD educational input is comparable to peer professions, AuD workforce growth is markedly less than peer professions ( Figs. 2 and 3 , Table 1 ) and even shows years of negative growth ( Figs. 3 , 5 , and 6 ). This means that AuD must have a greater departure rate than peer professions, where departure must include abandonment of the profession.

Table 4 , and extension of the work by Bray and Amlani, 6 displays the mean values derived from the CSD Education Survey report and BLS OEWS tables for the years 2009 to 2024. SLP, the larger profession, had more degrees awarded, a larger workforce, and greater year-to-year change in workforce numbers. For workforce IN, SLP at 5.9% was greater than AuD at 5.2%. For workforce OUT, SLP at −2.8% was less than AuD at −4.3%. For workforce growth (IN + OUT), SLP was greater at 3.1% than AuD at 0.9%. In summary, SLP was graduating more professionals with respect to their workforce size (5.9% vs. 5.2%), SLP was losing a smaller annual percentage of their workforce (−2.8% vs. −4.3%), and SLP workforce growth was 3.4 times AuD workforce growth (3.1% vs. 0.9%).

**Table 4:** Mean values (2009–2024) for audiology (AuD) and speech-language pathology (SLP) for entry-level degrees, workforce, year-to-year (Y2Y) change in workforce, workforce IN (degrees/workforce), workforce OUT (change minus degrees/workforce), and Y2Y IN + OUT

2010–2024	AuD mean	SLP mean
Degrees awarded	677	8,334
BLS OEWS workforce	12,906	141573
Y2Y workforce change	143	4,447
Workforce IN (%)	5.2%	5.9%
Workforce OUT (%)	−4.3%	−2.8%
Y2Y net: IN + OUT (%)	0.9%	3.1%

Abbreviations: BLS, Bureau of Labor Statistics; OEWS, Occupational Employment and Wage Statistics.

The linear regression lines in Fig. 6 display the long-term trends (2010–2024) for workforce IN and OUT for both AuD and SLP. The IN trend line for SLP (open circles with lines) is very stable over time at +6%. The IN trend line for AuD (filled circles) is changing, from about +4% in 2010 to +6% in 2024. The OUT trend line for SLP (open squares with lines) is slowly changing over time from −3 to −2%. The OUT trend line for AuD is changing (filled squares), from −6% in 2010 to about −3% in 2024. For AuD, a change was found for the combination of the IN and OUT trend lines, yielding net values of −2% for 2010 (4–6) to +3% for 2022 (6–3), where net values that are negative indicate a shrinking workforce and net values that are positive indicate a growing workforce.

The SLP IN line of +6% of the workforce (via education) and −2 to −3% OUT of the workforce (via retirement, death, and abandonment) for NET of +3 to +4% reinforce the previous observation that healthy professions, like SLP, can double their workforce over about a 25-year period. The changing AuD IN line of +3 to +6% and change of −6 to −3% in AuD OUT identify a profession that is not growing at a healthy rate, but that may be showing a long-term trend toward improvement. However, this trend must be interpreted with caution as the 2023 data again show a departure rate that exceed the graduation rate.

Utilizing information from Table 4 and Fig. 6 , SLP as a healthy workforce profession and AuD as an unhealthy workforce profession can be compared by using additional modeling. From Table 4 , the SLP aggregate IN + OUT value was +3.1% whereas the AuD aggregate was +0.9%. If departures from the profession are attributable to retirement, death, and abandonment, and if retirement and death rates are comparable for the two professions, the 2.2% difference between SLP and AuD must be attributed to an abnormally high abandonment rate within the AuD profession. Understanding why excessive abandonment is occurring and making changes to normalize the abandonment rate are the key issues to identify and resolve to increase the AuD workforce at a rate comparable to peer professions like OT, PT, SLP, and optometry.

## Limitations

There are limitations to this undertaking that warrant consideration. First, the profession lacks a single authoritative data source for enumerating the absolute size of the US AuD workforce. 12 Prior analyses have demonstrated substantial variability across federal datasets, professional association records, and provider registries, with estimates differing by several thousand audiologists depending on the source. 13 Administrative registries such as the National Provider Identifier (NPI) may overestimate active clinical supply because records are not automatically deactivated upon retirement or transition to non-clinical roles, whereas education- and employment-based datasets may undercount self-employed or nontraditional practitioners. 14 Although such discrepancies complicate point-in-time workforce enumeration, they do not materially affect the longitudinal trends or cross-profession comparisons reported here, which rely on internally consistent data sources.

Second, while the IN/OUT model presented in this paper provides evidence consistent with elevated departure rates in AuD relative to peer professions, precise individual-level attrition data remain difficult to establish from aggregate workforce and graduation records alone. Windmill and Freeman 14 reported attrition rates as high as 41% for audiologists, suggesting the departure problem identified here has prior empirical precedent—though the mechanisms driving abandonment specifically, as distinct from retirement and death, have not been directly measured in either study. Improved data collection on workforce entry, exit, and career duration will be essential for refining future workforce models, isolating the abandonment component, and developing evidence-based strategies to increase retention.

## Conclusion

Taken together, these findings characterize AuD as a doctoral-level profession with a stable and growing educational pipeline but constrained workforce growth relative to both population demand and peer health professions. Under current conditions, the profession risks widening access gaps and increasing reliance on alternative provider pathways. Importantly, the transition to the AuD as the terminal degree appears to have introduced a structural legacy effect on workforce capacity, the consequences of which persist decades after implementation. The opportunity state for AuD is, therefore, not limited to expanding enrollments; it lies in improving retention, strengthening utilization within healthcare systems (Article 3 on Labor, Revenue & Taxonomy), and aligning reimbursement and regulatory frameworks (Article 4, Nomenclature & Taxonomy) with the profession's training (Article 6, Evolving AuD Education), scope, and role within modern healthcare delivery (Article 7, Health Economics Perspective).

**Appendix A:** Bureau of Labor and Statistics (BLS) Standard Occupational Classification (SOC) grouped by degree requirement and mean workforce size (2024) for the healthcare professions shown in Figs. 1 (associate and bachelor's degrees) and 2 (master's and doctoral or professional degrees)

Healthcare professions by degree	BLS SOC	Workforce change (2012–2014)	2024 workforce size
**Associate's degree**			
Radiologic technologists and technicians	29–2034	1.16%	223,460
Dental hygienists	29–1292	1.28%	219,070
Respiratory therapists	29–1126	1.31%	136,420
Veterinary technologists and technicians	29–2056	3.89%	131,320
Diagnostic medical sonographers	29–2032	3.44%	86,460
Cardiovascular technologists and technicians	29–2031	1.65%	61,180
Magnetic resonance imaging technologists	29–2035	2.93%	41,530
Radiation therapists	29–1124	0.38%	18,700
Nuclear medicine technologists	29–2033	−1.64%	16,960
**Bachelor's degree**			
Registered nurses	29–1141	1.86%	3,282,010
Clinical laboratory technologists and technicians	29–2010	0.63%	343,040
Dietitians and nutritionists	29–1031	2.33%	76,570
Recreational therapists	29–1125	−1.86%	15,060
Exercise physiologists	29–1128	3.11%	8,110
**Master's degree**			
Nurse practitioners	29–1171	9.31%	307,390
Speech-language pathologists	29–1127	3.31%	178,790
Physician assistants	29–1071	5.31%	155,540
Occupational therapists	29–1122	3.16%	152,280
Nurse anesthetists	29–1151	3.33%	50,350
Athletic trainers	29–9091	2.90%	28,950
Orthotists and prosthetists	29–2091	2.28%	9,930
Nurse midwives	29–1161	4.20%	8,280
Genetic counselors	29–9092	5.26%	3,510
**Doctoral or professional degree**			
Pharmacists	29–1051	1.31%	328,870
Physical therapists	29–1123	2.23%	248,630
Dentists, general	29–1021	1.86%	113,490
Veterinarians	29–1131	3.10%	80,630
Optometrists	29–1041	3.14%	41,890
Chiropractors	29–1011	2.68%	37,630
Audiologists	29–1181	1.77%	14,730
Podiatrists	29–1081	0.46%	9,520

**Appendix B:** Ten professions and source for data on graduation rates by year (2017–2023) used to calculate graduate-to-workforce ratios

Profession	Source
Audiology/Speech-language pathology	Communication Sciences and Disorders (CSD) Education Survey (American Speech-Language-Hearing Association)
Chiropractic	Council on Chiropractic Education (CCE)
Nurse practitioner	American Association of Nurse Practitioners (AANP)
Occupational therapy	American Occupational Therapy Association (AOTA)
Optometry	Association of Schools and Colleges of Optometry (ASCO)
Orthotists and prosthetists	National Commission on Orthotic & Prosthetic Education (NCOPE)
Physician assistants	Physician Assistant Education Association (PAEA)
Physical therapy	Commission on Accreditation in Physical Therapy Education (CAPTE)
Podiatry	American Association of Colleges of Podiatric Education (AACPM)
